# Antioxidant potential of bitter cumin (*Centratherum anthelminticum *(L.) Kuntze) seeds in *in vitro *models

**DOI:** 10.1186/1472-6882-11-40

**Published:** 2011-05-20

**Authors:** V Ani, Kamatham A Naidu

**Affiliations:** 1 Department of Biochemistry and Nutrition, Central Food Technological Research Institute (Council of Scientific and Industrial Research), Mysore 570020, India

**Keywords:** Bitter cumin, *Centratherum anthelminticum*, polyphenolic compounds, antioxidants, DPPH, ABTS, reducing power, liposomes, oxidative DNA damage

## Abstract

**Background:**

Bitter cumin (*Centratherum anthelminticum *(L.) Kuntze), is a medicinally important plant. Earlier, we have reported phenolic compounds, antioxidant, and anti-hyperglycemic, antimicrobial activity of bitter cumin. In this study we have further characterized the antioxidative activity of bitter cumin extracts in various in vitro models.

**Methods:**

Bitter cumin seeds were extracted with a combination of acetone, methanol and water. The antioxidant activity of bitter cumin extracts were characterized in various *in vitro *model systems such as DPPH radical, ABTS radical scavenging, reducing power, oxidation of liposomes and oxidative damage to DNA.

**Results:**

The phenolic extracts of bitter cumin at microgram concentration showed significant scavenging of DPPH and ABTS radicals, reduced phosphomolybdenum (Mo(VI) to Mo(V)), ferricyanide Fe(III) to Fe(II), inhibited liposomes oxidation and hydroxyl radical induced damage to prokaryotic genomic DNA. The results showed a direct correlation between phenolic acid content and antioxidant activity.

**Conclusion:**

Bitter cumin is a good source of natural antioxidants.

## Background

In living systems, reactive oxygen species (ROS) constitute most important free radicals. ROS include not only oxygen radical, but, also some non radical derivatives of oxygen like H_2_O_2 _[1]. ROS play a positive role in energy production, phagocytosis, and regulation of cell growth, cell signaling and synthesis of biologically important compounds. Oxidative stress is the result of an increased ROS production and/a decrease in their elimination. Based on the fact that ROS are dangerous for cells, tissues and organs it has been inferred that oxidative stress is the cause for number of disorders, including atherosclerosis, neural degenerative disease, inflammation, cancer and ageing [2-4]. The physiological role of antioxidants is to prevent damage to cellular constituents arising as a consequence of chemical reactions involving free radicals [5,6]. The use of synthetic antioxidants in food has its beginning in the late 1940's when BHA was found to be effective antioxidant in fatty foods and toxicological studies proved it safe for food use. But, later there are serious concerns over the side effects of these synthetic antioxidants due to their carcinogenic potential [7,8]. As a result there has been a general desire to replace the synthetic food additives with natural antioxidants [9,10].

Phytochemicals are non-nutritive plant chemicals that have protective or disease preventive properties. There are thousands of phytochemicals falling into different groups and one of the most studied groups is phenolics. Plant phenolics are multifunctional and can act as reducing agents, metal chelators and singlet oxygen quenchers. Many studies have shown that phenolics are of great value in preventing the onset and/progression of many human diseases [11-13]. Therefore over the past few years a number of medicinal and food plants are extensively investigated for the presence and activity of polyphenols and other antioxidants [14-16]. There are several methods to determine the antioxidant activity of a biological sample each with its own advantages and disadvantages. These tests enable us to examine the possibility of a given biological sample could act as an antioxidant in one or more ways *in vivo *or in food substances.

*Centratherum anthelminticum *(L.) Kuntze (bitter cumin) is a member of Asteraceae family of the flowering plants. The seeds have a hot sharp taste; acrid, astringent to the bowls, antihelmintic; cure ulcers, used in skin diseases, leucoderma and fevers. Two novel and two known steroids were isolated respectively from benzene: acetone and ethanolic extracts of the seeds of *C. anthelminticum *[17]. The plant has reported to possess antimicrobial [18], antifilarial [19,20], post-coital anti-implantation [21] and insecticidal activities [22]. Earlier we have reported an array of phenolic compounds, antioxidant activity in few model systems, antimicrobial and anti-hyperglycemic activity of bitter cumin [23,24]. The present paper describes antioxidant studies with an emphasis on various *in vitro *antioxidant model systems.

## Methods

### Chemicals

1,1-Diphenyl -2-picryl hydrazyl (DPPH), 2,2 azinobis-3-ethyl benzothiazoline-6-sulfonic acid (ABTS), Butylated hydroxyl anisole (BHA), Ascorbic acid, α-Tocopherol, agarose, xylene cyanol, bromophenol blue, ethidium bromide, thiobarbituiric acid and tannic acid were purchased from Sigma chemicals (MO, USA). Bacillus genomic DNA was obtained from the Food Microbiology department of CFTRI. Folin-Ciocalteau reagent, was purchased from Sisco research laboratories (Mumbai, India). Ammonium molybdate, trichloroacetic acid, potassium ferricyanide and ferric chloride were purchased from Qualigens. All other chemicals and solvents used are of analytical grade.

### Plant material

The plant material was purchased from local market and authenticated by National Institute of Science Communication and Information Resources, New Delhi.

### Extraction

The seeds were hand sorted to remove stones and plant debris. The powdered seeds were defatted for 8 hours in Soxhlet's apparatus with hexane. The defatted powder was extracted with methanol:acetone:water (7:7:6) which is subsequently hydrolyzed with 2N HCl and extracted into ethyl acetate and named as Aqueous Methanol Acetone Extract -(AMAECA). The defatted powder was extracted with 80% methanol and termed as Aqueous Methanol extract (AMECA). The defatted bitter cumin seed powder was extracted with water and named as aqueous extract- (AECA) (1:10 w/v × 3) under continuous stirring at ambient temperature. The organic solvents were removed under vacuum in a rotavapour and water was removed by freeze drying. The solid extract obtained was stored at 4°C until use.

### Estimation of total phenols

Total phenol content of the AMAECA, AMECA and AECA were estimated by using Folin-Ciocalteu reagent [25]. 0.5 mL of the sample dissolved in MeOH was incubated with 2.5 mL of 10% FC reagent for 2 min at ambient temperature. To this 2.0 mL of 7.5% Na_2_CO_3 _was added and incubated for another 1 hour at ambient temperature. The absorbance of the color developed was measured at 765 nm against blank developed with 0.5 mL of solvent using a Shimadzu UV-Visible spectrophotometer (Model- 2100). The total phenolic content was expressed as tannic acid equivalents (TAE) in μg/mg of the extract, using a standard curve generated with tannic acid. Similarly, the total phenol content was also expressed as gallic acid equivalents (GAE) using a standard curve generated with gallic acid as standard.

### In vitro antioxidant activity of bitter cumin extracts

#### DPPH radical scavenging assay

The antioxidant activity of different extracts of BITTER CUMIN and standard BHA was measured in terms of hydrogen donating or radical scavenging ability using the stable DPPH^• ^method Brand Williams et al. 1995 [26]. Briefly 2 ml of 100 μM methanolic solution of DPPH^• ^was incubated with 100 μl of bitter cumin extracts or BHA and incubated for a period of 20 minutes at ambient temperature. At the end of incubation period the OD was measured using a UV-Visible spectrophotometer at 517 nm against MeOH blank. 100 μl solvent was added to control test tube under the same conditions. The percentage of scavenging or quenching of DPPH radicals (Q) by bitter cumin and BHA was calculated using the following formula.

Where Ao is the absorbance of the control tube Ac is the absorbance of the tube with 'c' concentration of sample [27]. All the experiments were performed in triplicates.

#### ABTS^•+ ^scavenging assay

Generation of ABTS^**•+ **^radical [28] forms the basis of one of the spectrophotometric methods that have been applied to the measurement of the total antioxidant activity of various substances. The experiments were carried out using an improved ABTS^•+ ^decolorization assay [29]. ABTS radical cation (ABTS^•+^) was produced by reacting ABTS stock solution with 2.45 mM potassium persulfate (final concentration) and allowing the mixture to stand in the dark at room temperature for 12-16 h before use. The ABTS^•+ ^solution was diluted to an absorbance of 0.7 ± 0.05 at 734 nm (Shimadzu UV-Vis Spectrophotometer) with ethanol. To one ml of ABTS^•+ ^solution different concentrations of bitter cumin extracts/BHA were added. Absorbance was recorded at 1 min interval up to 7 minutes at 734 nm. All the experiments were performed in triplicates.

#### Phosphomolybdenum reducing assay

This assay is based on the reduction of Mo (VI) to Mo (V) by the sample analyte and the subsequent formation of a green phosphate/Mo (V) complex at acidic pH [30]. The reagent solution consists of 0.6 M H_2_SO_4_, 28.0 mM sodium phosphate and 4.0 mM ammonium molybdate. An aliquot of 0.1 ml of sample was combined with 1 ml of reagent solution. The tubes were capped and incubated in a thermal block at 95°C for 90 min. After the samples had cooled to room temperature, the absorbance of the aqueous solution of each was measured at 695 nm against a blank. The blank solution contained 1 ml of reagent solution and the solvent used for the sample, and it was incubated under the same conditions as the rest of the samples. All the experiments were performed in triplicates. The antioxidant capacity was expressed as equivalents of ascorbic acid. (μg/g extract).

#### Ferricyanide reducing assay

The reductive potential of the extract was determined according to the method of Oyaizu et al. 1986 [31]. Different concentrations of sample in 0.5 ml of MeOH were mixed with 2.5 ml of Phosphate buffer (0.2 M, pH 6.6) and 2.5 ml of 1% potassium ferricyanide. The mixture was incubated for 20 minutes at 50° C. At the end of the incubation, 2.5 ml of 10% trichloroacetic acid was added to the mixture and centrifuged at 5000 rpm for 10 minutes. The upper 2.5 ml layer was mixed with 2.5 ml of distilled water and 0.5 ml of 0.1% ferric chloride, and the absorbance was measured at 700 nm [32]. A higher absorbance of the reaction mixture indicated greater reducing power. Ascorbic acid and BHA were used as a positive control.

#### Liposome oxidation assay

The antioxidant activity of the extracts of bitter cumin and α-tocopherol in a liposome model system was determined according to the method of Duh and Yen 1997 [33]. Egg lecithin (300 mg) was sonicated with 30 ml phosphate buffer (10 mM, pH 7.4) in an ultrasonic sonicator for 30 min to ensure proper liposome formation. Bitter extracts and standards were mixed with the sonicated solution (0.5 ml, 10 mg/ml) and incubated for 10 min. at room temperature. The oxidation of liposomes was initiated by adding FeCl_3 _(0.5 ml, 400 mM), and ascorbic acid (0.5 ml, 400 mM). The antioxidative action was measured by the method of Buege and Aust 1978 [34]. The absorbance of the samples was determined at 535 nm after incubation for 1 h at 37°C. The results were expressed as nmol of malondialdehyde (MDA) formed per mg lipid and was calculated by using an extinction coefficient of 1.56 × 10^5 ^M^_1 ^cm^_1^.

#### Oxidative DNA damage assay

Bacterial genomic DNA (2 μg) in phosphate buffered saline was incubated with different concentrations of bitter cumin and BHA for 15 minutes at ambient temperature. Oxidation was induced by treating DNA with 1 mM FeSO_4 _and 10 mM ascorbic acid. Positively controlled reaction was not treated with bitter cumin or oxidative stress and negatively controlled reaction mixture contained FeSO_4 _and ascorbic acid without any pretreatment with CA. The final reaction volume was 9 μl and the reaction mixture was incubated for 1 hour at 37°C. The reaction was stopped by adding 3 μl loading buffer (xylene-cyanol, 0.25%; bromophenol blue, 0.25%; and glycerol, 30%) and 9 μl of the reaction mixture was loaded on to an agarose gel (1%). The gel was run in TAE buffer initially for 1 h at 40 V followed by 2 hours run at 60 V. The gel was stained with ethidium bromide (1 μg/ml). DNA was visualized and photographed by a digital imaging system (Hero lab, GMBH, Germany).

### Statistical analysis

All the experiments were done in triplicates and expressed as mean ± S.E.M. The differences in mean values were tested using one-way analysis of variance (ANOVA) and Duncan's multiple range test (DMRT) was used to determine the significant differences amongst the test materials. Differences were considered to be significant at p ≤ 0.05.

## Results and Discussion

### Total phenol acids

**Figure 1:**
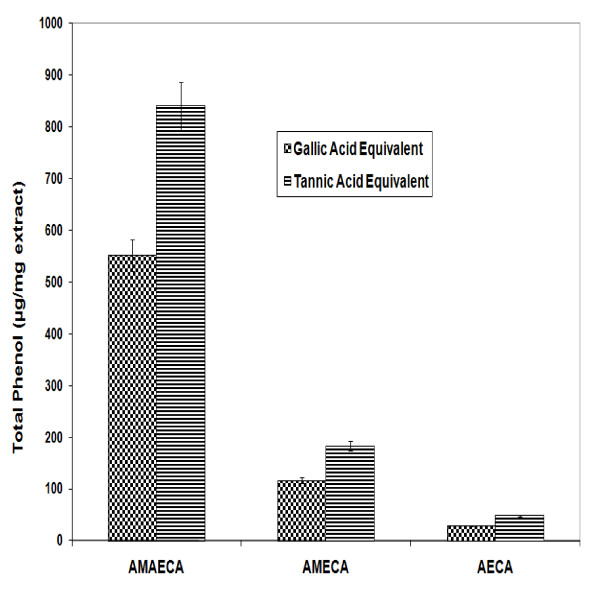
**Total phenol content of different extracts of bitter cumin**. Values are mean ± S.E.M. of three experiments.

Phenols, are a major group of plant metabolites, have profound importance due to their biological properties. The number, type and concentrations of phenols in plants exhibit extreme diversity [35,36]. Extraction method and solvent choice are generally critical and no single solvent provides optimum recovery of all phenols. Often a combination of solvents will provide optimum recovery of all phenols or at least a limited range of phenols and with dried materials alcoholic solvents presumably rupture cell membranes and enhance the extraction of endocellular materials [37]. The total phenol content of Bitter cumin seeds was extracted with aqueous methanol-acetone (methanol:acetone:water 7:7:6), 80% MeOH and water. The total phenol content was estimated using Folin-Ciocalteu reagent and the values are expressed as gallic acid [25] and tannic acid equivalents (Figure 1). Significant variation in the phenolic content was observed in different extracts of bitter cumin (p ≤ 0.05). Aqueous methanol acetone extract showed highest total phenol content of 551.8 ± 30.8 μg GAE/mg or 840.8 ± 46.9 μg TAE/mg) and aqueous extract showed lowest total phenolic content of 29.2 ± 1.0 μg GAE/mg or 48.6 ± 1.6 μg TAE/mg.

### Radical scavenging activity of bitter cumin phenols

**Figure 2:**
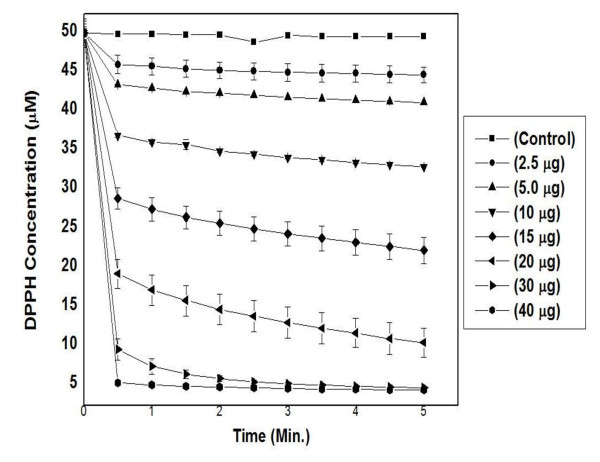
**Kinetics of DPPH^• ^scavenging by different concentrations of bitter cumin extract (AMAECA)**. Values are mean ± S.E.M. of three experiments.

**Figure 3:**
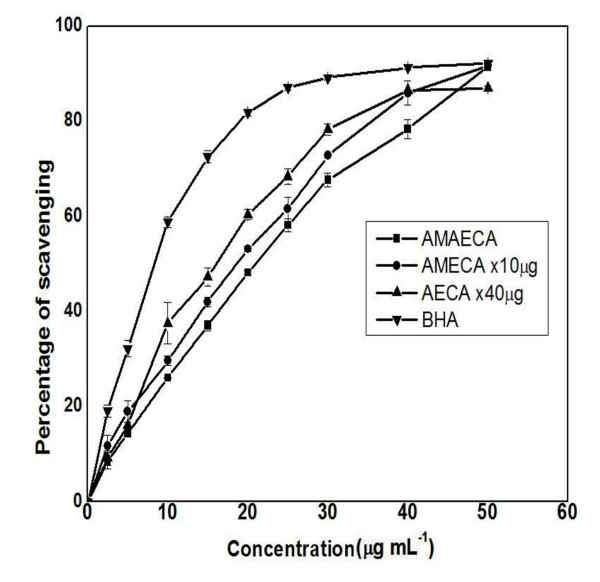
**DPPH radical scavenging activity of different extracts of bitter cumin**. Values are mean ± S.E.M. of three experiments.

In the DPPH test, the stable, nitrogen centered, coloured, DPPH^• ^free radical is reduced either by hydrogen donor or antioxidant to a non-radical DPPH-H and the decrease in colour of DPPH radical is monitored over a time period [38]. The kinetics of DPPH^• ^radicals scavenging activity increased with an increase in the concentration of cumin extract as shown in Figure 2. Addition of bitter cumin extract (AMAECA) showed a sharp drop in DPPH colour intensity, indicating high antioxidant activity in quenching DPPH radicals during the first 30 seconds of the reaction followed by a logarithmic decay. The DPPH^• ^scavenging potential of various bitter cumin extracts and BHA are presented in Figure 3. The correlation coefficient between dose and scavenging percentage are 0.8520, 0.9881, 0.9801 and 0.9475 respectively for BHA, AMAECA, AMECA and AECA. BHA, the synthetic antioxidant showed highest DPPH^• ^scavenging activity among the tested samples with an IC_50 _value of 8.2 ± 0.24 μg. Among various extracts of bitter cumin AMAECA showed highest DPPH^• ^scavenging activity (IC_50 _20.8 ± 0.18 μg) (Table 1), followed by AMECA (IC_50 _191.7 ± 9.14 μg) and AECA (IC_50 _639.2 ± 20.91 μg).

**Table 1:** Antiradical activity of bitter cumin extract (AMAECA)

Antiradical assay	Radical donated/scavenged	IC _50 _values (μg of AMAECA)
Phosphomolbdenum reducing power	Electron donation	0.31
Ferricyanide reducing Power	Electron donation	0.20
ABTS radical assay	ABTS^•+^	8.3
DPPH radical assay	DPPH^•^	20.8
Liposomal lipid peroxidation	OH^•^	14.3

**Figure 4:**
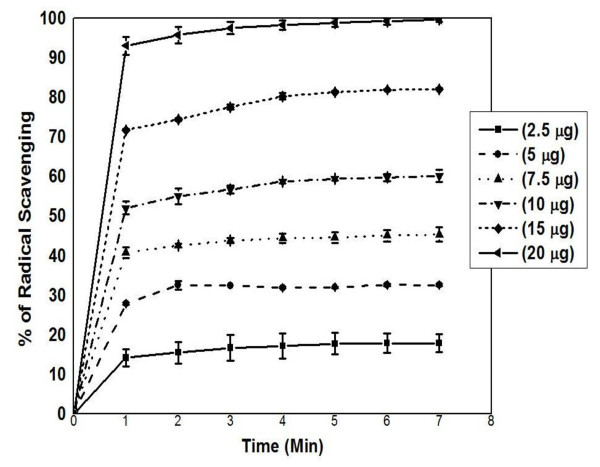
**Kinetics of ABTS^•+ ^scavenging of by AMAECA**. Values are mean ± S.E.M. of three experiments.

ABTS test is based on the formation of stable, blue-green coloured ABTS^**•+ **^radical for the measurement of the total antioxidant activity of both water-soluble and lipid-soluble antioxidant compounds [39,40]. In the presence of an antioxidant the colour production will be suppressed to an extent proportional to the concentration of antioxidants. The kinetics of ABTS^**.+ **^scavenging activity of bitter cumin extract (AMAECA) is presented in Figure 4. ABTS^**.+ **^scavenging by AMAECA occurred during first 1 min of the reaction indicating potent antioxidant activity of bitter cumin. IC_50 _value for quenching ABTS^**.+ **^was found to be 8.3 (Table 1), 47.0 and 68.0 μg for bitter cumin extracts AMAECA, AMECA and AECA, respectively. Thus bitter cumin extracts showed significant scavenging of DPPH and ABTS radical at μg concentrations.

### Reducing power of bitter cumin phenolics

**Figure 5:**
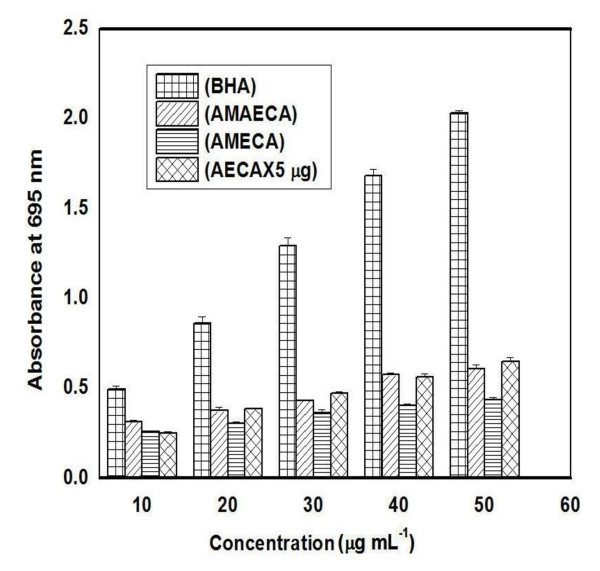
**Phosphomolybdenum reducing activity of bitter cumin extracts and BHA**. Values are mean ± S.E.M. of three experiments.

The reducing power of a compound is associated with electron donating capacity and serves as an indicator of antioxidant activity [41,42]. Phosphomolybdenum reducing power increased with the concentration of bitter cumin extracts as shown in Figure 5. There was a direct relationship between the dose and absorbance. The correlation coefficient of dose versus absorbance was 0.9994, 0.9812, 0.9943 and 0.9957 for BHA, AMAECA, AMECA and AECA respectively. The highest reducing potential was shown by BHA followed by AMAECA, AMECA and AECA. The IC_50 _value for AMAECA was found to be 0.31 μg (Table 1).

**Figure 6:**
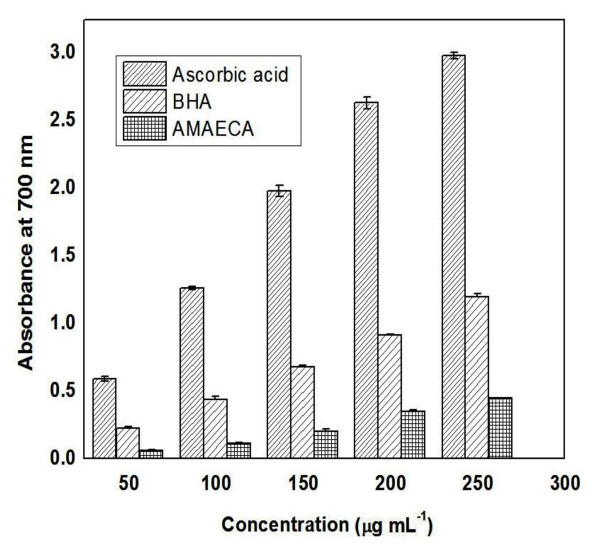
**Fe**^**3+**^**reducing power of bitter cumin extract (AMAEC), ascorbic acid and BHA**. Values are ± S.E.M. of three experiments.

Further, the ability of bitter cumin extracts to reduce Fe^3+ ^to Fe^2+ ^was determined and compared with that of a standards BHA and ascorbic acid. All the three bitter cumin extracts showed some degree of electron donating capacity and reduced Fe^3+ ^to Fe^2+^. All the bitter cumin extracts exhibited lower activity than the standards (Figure 6). The correlation coefficient between dose and absorbance was 0.9937, 0.9988 and 0.9878 respectively for Ascorbic acid, BHA and AMAECA. The IC_50 _value for AMAECA was found to be 0.20 μg (Table 1).

### Inhibition of liposome oxidation activity by bitter cumin phenols

**Figure 7:**
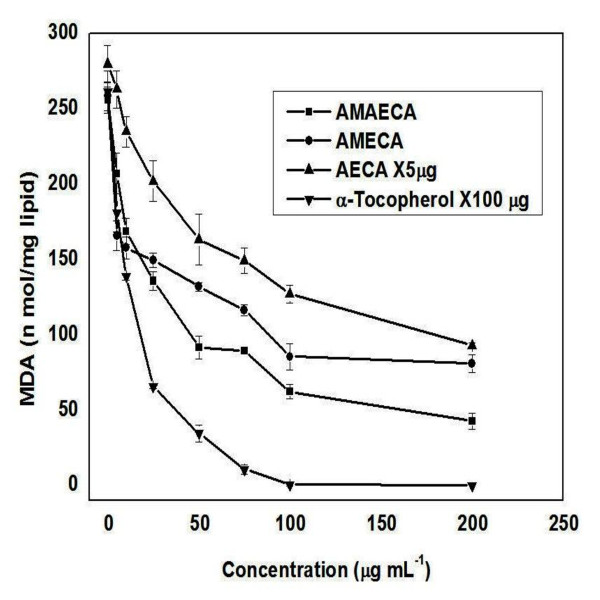
**Inhibition of liposomal peroxidation by bitter cumin extracts and α-tocopherol**. Values are mean ± S.E.M. of three experiments.

Cellular membranes, which contain abundant phospholipids, such as phosphatidylcholine (lecithin), are major targets of free radicals which induce lipid peroxidation and thereby cause malfunctioning of membranes by altering membrane fluidity and membrane bound enzyme and receptor functions [43]. Further, several toxic byproducts of the peroxidation can damage other biomolecules including DNA away from the site of their generation [44,45]. Liposomes are spherical, self closed vesicles of colloidal dimensions, in which (phospho) lipid bilayer sequesters part of the solvent, in which they freely float, into their interior [46]. The use of liposomes appears to be the most promising method of assessing antioxidant properties relevant to human nutrition, since these systems allow investigations of the protection of a substrate by an antioxidant in a model biological membrane or lipoprotein. Figure 7 shows the inhibition of phospholipid peroxidation in presence of bitter cumin extracts and α-tocopherol. AMAECA showed highest inhibitory activity and AECA showed lowest activity. All the extracts of bitter cumin showed higher inhibitory activity against phospholipid peroxidation compared to the standard antioxidant α-tocopherol. The bitter cumin extracts AMAECA, AMECA, AECA were found to be 41, 24 and 3 times more potent than α-tocopherol in inhibiting liposome oxidation, respectively. Thus, the efficiency was in the order of AMAECA > AMECA > AECA > α-tocopherol. The highest correlation between dose and antioxidant activity was shown by AECA (0.9041) and others showed a correlation coefficient of 0.8288 by AMAECA, 0.7560 by AMECA and 0.7364 by α-tocopherol, respectively.

### Inhibition of oxidative damage to DNA by bitter cumin phenols

**Figure 8:**
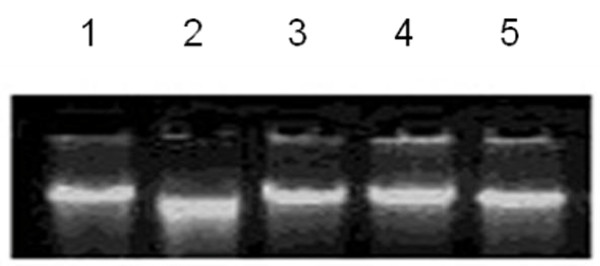
**Protective effect of AMAECA on oxidative damage to genomic DNA of Bacillus**. Lane 1: Native genomic DNA of Bacullus; Lane 2: DNA + 1.0 mM FeSO_4 _+10.0 mM Ascorbic acid; Lane 3: DNA + 1.0 mM FeSO_4 _+10.0 mM Ascorbic acid + 1.0 mg BHA; Lane 4: DNA + 1.0 mM FeSO_4 _+10.0 mM Ascorbic acid + 1.0 mg AMAECA; Lane 5: DNA + 1.0 mM FeSO_4 _+10.0 mM Ascorbic acid + 2.5 mg AMAECA

The most detrimental of the free radicals formed in biological systems is the hydroxyl radical that causes enormous damage on biomolecules of the living cells [47]. DNA is susceptible to oxidative damage and hydroxyl radicals oxidize guanosine or thymine to 8-hydroxyl-2- deoxyguanosine and thymine glycol which change DNA and lead to mutagenesis and carcinogenesis [48]. DNA damage results in mutations and altered cell functions. A large proportion of these mutagenic steps precede carcinogenic events. In this study, hydroxyl radicals generated by Fenton reaction were found to induce DNA strand breaks in prokaryotic genomic DNA. Earlier, we reported protection of calf thymus DNA and plasmid DNA (pUC18) against the oxidative damage by bitter cumin extract [23]. In this study, AMAECA was tested for its potential to inhibit the DNA damage induced by hydroxyl radical in prokaryotic genomic DNA. AMAECA at 1.0-2.5 μg offered complete protection to DNA damage in prokaryotic genomic DNA (Figure 8). Thus, the hydroxyl radical quenching ability of extracts of bitter cumin could be responsible for the protection against oxidative damage to DNA.

## Conclusion

The results from various free radical scavenging systems revealed that bitter cumin extracts were strong antioxidants, with different magnitudes of potency in scavenging different ROS at microgram concentrations. The antioxidant activity of bitter cumin significantly correlated with total phenol content of bitter cumin extract. The phenol extract of bitter cumin contained an array of phenolic compounds which may be responsible for its antioxidant activity.

## Competing interests

The authors declare that they have no competing interests.

## Authors' contributions

AV conducted experiments on antioxidant activity of bitter cumin. KAN participated in design of the study and preparation of the manuscript. All the authors read and approved the final manuscript.

## Pre-publication history

The pre-publication history for this paper can be accessed here:

http://www.biomedcentral.com/1472-6882/11/40/prepub
